# The larva and prepupa of *Eupareophora exarmata* (Thomson, 1871) (Hymenoptera, Tenthredinidae)

**DOI:** 10.3897/BDJ.3.e7147

**Published:** 2015-11-25

**Authors:** Andrew David Liston, Marko Prous, Josef Bücker

**Affiliations:** ‡ Senckenberg Deutsches Entomologisches Institut, Müncheberg, Germany; § Department of Zoology, Institute of Ecology and Earth Sciences, University of Tartu, Tartu, Estonia; | In der Geweke 40, Hagen, Germany

**Keywords:** *Eupareophora exarmata*, sawfly, larva, prepupa, Rosaceae, ash species, *
Fraxinus
*, Germany

## Abstract

**Background:**

Of the two known *Eupareophora* species, more is known about the larva  and bionomics of the Nearctic *E. parca*, than the rarely recorded West Palaearctic *E. exarmata*.

**New information:**

The last instar larva and prepupa of *E. exarmata* is illustrated and briefly described. In Germany its host is *Fraxinus excelsior*.

## Introduction

*Eupareophora exarmata* (Thomson, 1871) is a rarely recorded sawfly species with a wide West Palaearctic range extending from southern Sweden (type locality: Lund; Thomson 1871) to Spain (Taeger et al. 2006) and reaching the Caucasus in the East (Supatashvili et al. 1972). The only other known *Eupareophora* species is the Nearctic *E. parca* (Cresson, 1880). While the larva, hosts and biology of *E. parca* are described and discussed by Smith 1969 and Williams 2007, very little such information is available on *E. exarmata*. During recent years several distinctive sawfly larvae were found at a locality in north-west Germany, associated with ash (*Fraxinus excelsior*). Although adults were not reared, examination of the morphology of the larvae and a comparison of a mitochondrial DNA sequence from one prepupa with sequences of other Tenthredinidae, indicated beyond reasonable doubt that they belong to *E. exarmata*. We hope that the short, illustrated descriptions of the mature larva and prepupa provided below may lead to increased recording of this sawfly species.

## Materials and methods

Material examined: *Eupareophora exarmata*

Germany, Nordrhein-Westfalen, Hagen-Hohenlimburg, 51.32099, N 7.57673 E, 172 m. a.s.l., all records by J. Bücker: 31.5.2009, 2 larvae about 30-50 cm above soil level on trunk of approximately 50 year old *Fraxinus excelsior*; 24.5.2011, 5 larvae, on same tree but 50-120 cm above soil level; 2.6.2012, 1 larva on metal fence post. On the first two dates larvae were photographed but not collected. The last larva was collected. It moulted to the prepupal stage within 24 hours of being found. In anology with the observations by Williams 2007 on *E. parca*, it was probably 5th instar when collected. The cast larval skin was dry-mounted, and the prepupa conserved in ethanol. These specimens are deposited in the Senckenberg Deutsches Entomologisches Institut, Müncheberg.

Molecular methods

To assess the phylogenetic position of *Eupareophora exarmata* within Tenthredinidae, full or partial (at least 1119 bp) cytochrome c oxidase I gene (COI) sequences were sequenced from the prepupa of the putative *E. exarmata* (GenBank accession KT964163) as well as various other tenthredinids as described previously (Prous et al. 2011; Prous and Heidemaa 2012). Some additional COI sequences representing broad diversity of Tenthredinidae, Cimbicidae, Diprionidae, and *Heptamelus*, and which were at least 1000 bp long, were downloaded from NCBI nucleotide database (http://www.ncbi.nlm.nih.gov/nucleotide). The downloaded sequences were published previously by Prous et al. 2011, Boevé et al. 2013, Wei et al. 2015a, Wei et al. 2015b, Malm and Nyman 2014, and Song et al. 2015. Only the downloaded COI sequence of *Eupareophora parca* was shorter than others (KF528474; 862 bp), because this was the only *Eupareophora* sequence publicly available. Representatives of Cimbicidae, Diprionidae, and *Heptamelus* were used as an outgroup, as these are the closest relatives of Tenthredinidae (Malm & Nyman 2015). PhyML (Guindon et al. 2010) was used to estimate a maximum likelihood tree of Tenthredindae COI sequences. GTR+G model was employed and node support values were evaluated based on 500 bootstrap replicates using PhyML online version (http://www.atgc-montpellier.fr/phyml/). Following the results of Malm and Nyman 2014, *Heptamelus* was used to root the tree. Newly obtained sequences have been deposited in the GenBank (NCBI) database (accession numbers KT964153-KT964167).

## Taxon treatments

### Eupareophora exarmata

(Thomson, 1871)

#### Materials

- **Type status:** Other material. **Occurrence:** catalogNumber: DEI-GISHym19361; individualCount: 1; lifeStage: prepupa; occurrenceStatus: present; preparations: whole animal (ethanol) and larval exuvia; disposition: in collection; **Taxon:** scientificName: *Eupareophora exarmata* (Thomson, 1871); kingdom: Animalia; phylum: Arthropoda; class: Insecta; order: Hymenoptera; family: Tenthredinidae; genus: Eupareophora; specificEpithet: exarmata; scientificNameAuthorship: Thomson, 1871; nomenclaturalCode: ICZN; taxonomicStatus: accepted; **Location:** continent: Eurasia; country: Germany; countryCode: DE; stateProvince: Nordrhein-Westfalen; locality: Hagen-Hohenlimburg; decimalLatitude: 51.32099; decimalLongitude: 7.57673; **Identification:** identifiedBy: Andrew Liston; **Event:** year: 2012; month: 6; day: 2; **Record Level:** type: PhysicalObject; language: en; institutionCode: SDEI; basisOfRecord: PreservedSpecimen

#### Description

**Sequencing results**

**Figure 1.**
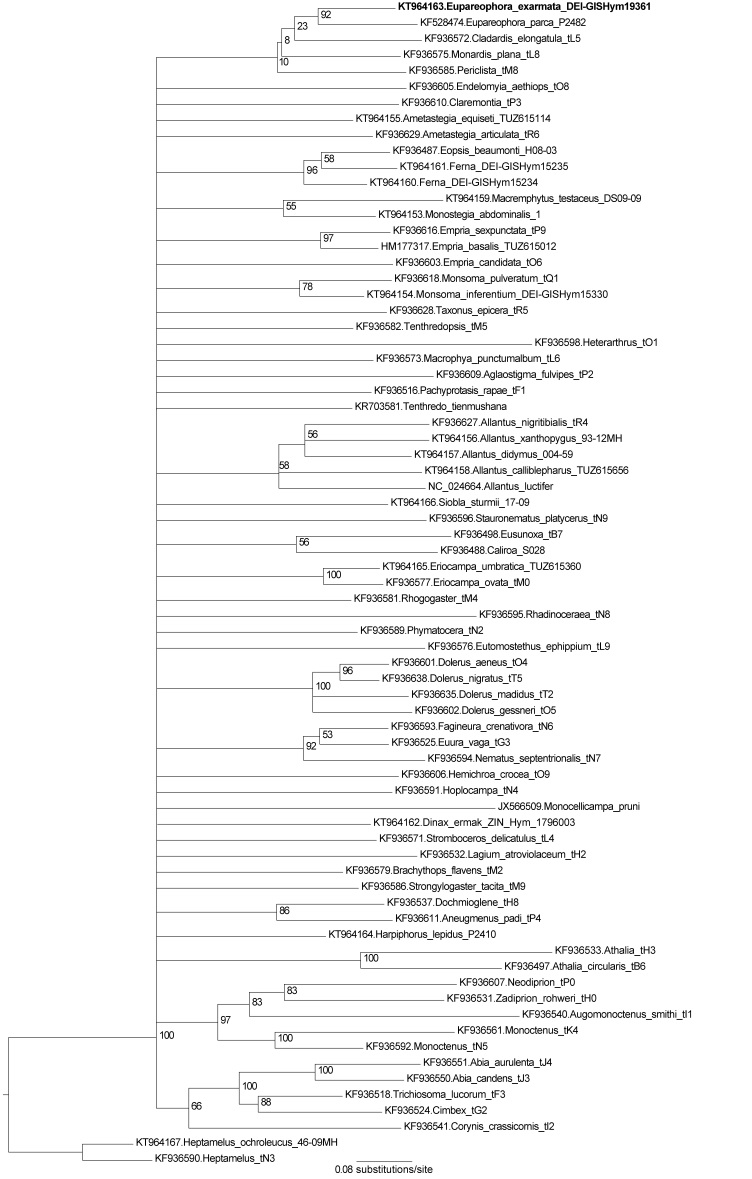
Maximum likelihood tree of COI (1078 bp) sequences of Tenthredinidae. Numbers at nodes are bootstrap proportions (%) derived from 500 pseudoreplicates. Nodes receiving bootstrap support less than 50% have been collapsed, except for those closest to *Eupareophora*.

Phylogenetic analyses of 1078 bp of COI sequences showed with strong statistical support (bootstrap proportion 92%) that the closest relative of the putative *Eupareophora exarmata* prepupa is the Nearctic *E. parca* (Fig. 1), from which it nevertheless differs significantly at the sequence level, by 10.8%. The tree is otherwise poorly resolved, because of the limited amount of sequence data used. Closest relatives of the genus *Eupareophora* on the tree are *Cladardis*, *Monardis*, and *Periclista*, although without statistical support (Fig. 1). A strongly supported clade of the latter three genera was found by Malm and Nyman 2014 using 8 nuclear and one mitochondrial (COI) protein coding genes, suggesting that *Eupareophora* (which Malm & Nyman 2015 did not sample) might also belong there.

**Hosts**

Liston 1995 stated that the hosts of *Eupareophora exarmata* are *Rosa* species (Rosaceae) and that the larvae bore in shoots. Although not cited by Liston, this statement was based on a record by Reichert 1933: “mit Larven von *Ardis brunniventris* eingetragene Rosenzweige ergaben 13.2.18. im geheizten Zimmer 1 #w, det Enslin”. In view of the morphological similarity of adult *M. plana* (whose hosts are *Rosa* spp.: Scheibelreiter 1973) and *E. exarmata*, it seems likely that Enslin misidentified the specimen. Zhelochovtsev [Zhelohovcev] 1988) mentioned under *E. exarmata* simply “on ash” [translated] (*Fraxinus* sp., Oleaceae). Probably this information is based on original observations made by Supatashvili et al. 1972 al. (1972) in Georgia, who reared adults from larvae. Adults were examined by Zhelochovtsev and determined as *E. exarmata*. The larva is very briefly described by Supatashvili et al. 1972 [translated]: “Larva grey coloured, body covered with awl-shaped processes”. These authors also record “ash” as the host, but do not mention which *Fraxinus* species was involved. Apart from *Fraxinus excelsior* L., some other ash species occur in Georgia, such as *F. angustifolia* Vahl subsp. *oxycarpa* (M. Bieb. ex Willd.) Franco & Rocha Afonso (USDA 2012). The recent German records indicate that *F. excelsior* L. is a host. As far as we are aware, the publication by Supatashvili et al. 1972 is unique in referring to *E. exarmata* as a pest. Their observations were made in stands of planted ash. It is noteworthy that the occasional reports of defoliation caused by *E. parca* in North America involve “planted ash species [..] in urban settings” (Williams 2007), although D. R. Smith (personal communication) states that it is also fairly common throughout the eastern deciduous forests.

**Figure 2.**
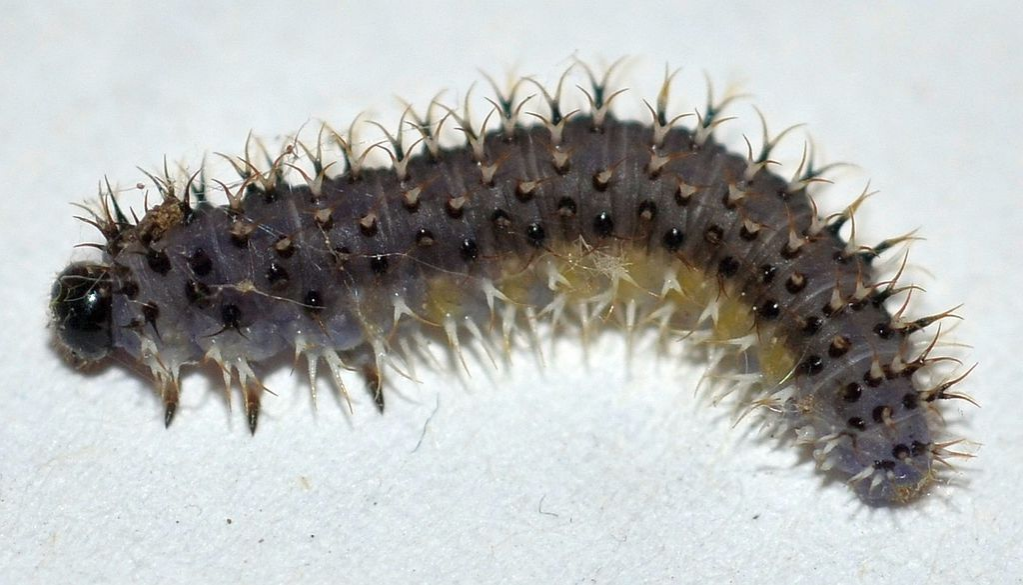
*Eupareophora exarmata*, larva, last feeding instar; Germany, Hagen-Hohenlimburg. Photos: J. Bücker.

**Figure 3.**
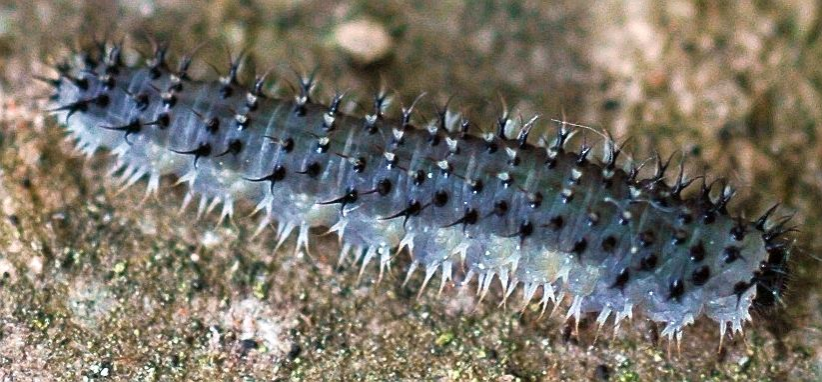
*Eupareophora exarmata*, larva, last feeding instar.

**Figure 4.**
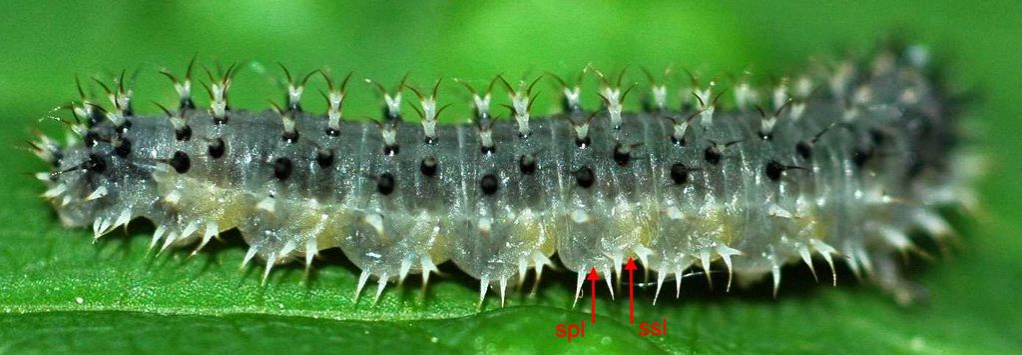
*Eupareophora exarmata*, larva, last feeding instar; spl=surpedal lobe, ssl=subspiracular lobe.

**Description of mature larva** (Figs 2, 3, 4).

Terminology follows Viitasaari 2002, with notation of annulets of abdominal segments according to Vikberg and Nuorteva 1997, i.e. annulet 3 bears the spiracle.

Length: approximately 15 mm.

Head completely black except for pale mouthparts. Ground colour of trunk above spiracular line grey; whitish below this, with yellow tinge on abdominal segments 1-8. Cuticular processes (hereafter: spines) above spiracles located on more or less black glandubae. Above spiracles, on thorax, most spines entirely black; on abdomen all supraspiracular spines blackish above fork, whitish below this; the outermost of each dorsal pair of spines darker. All subspiracular spines paler than more dorsal ones; apically at most pale brown, and if located on glandubae, then these also completely pale.

Antenna with 5 articles. Clypeus with 2 setae. Thoracic leg with 5 articles. The 4 most dorsal and anterior spines on thorax are trifid. Prolegs on abdominal segments 2-8 and 10. Abdominal segments 1-9 with 5 dorsal annulets. Annulet 3 with 2 supraspiracular bifid spines. Annulet 5 with 3 bifid spines: 2 supraspiracular and 1 on spiracular line. Abdominal segment 10 without spine on midline. Subspiracular lobe with two spines; anterior one bifid, other simple. Suprapedal lobe with two simple spines.

**Figure 5.**
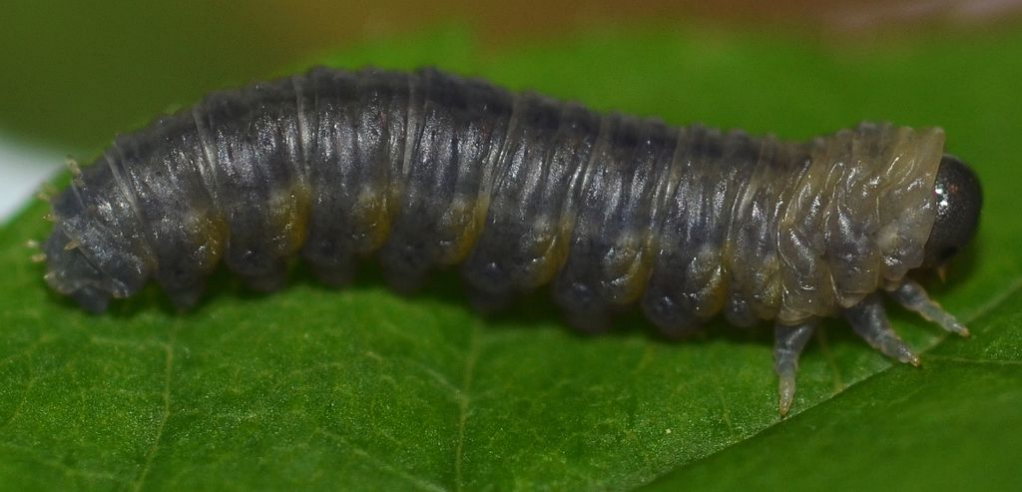
*Eupareophora exarmata*, Germany, Hagen-Hohenlimburg; prepupa. Photo: J. Bücker.

**Description of prepupa** (Fig. 5).

Length: approximately 13 mm.

Head grey above; yellowish on and around mouthparts. Thorax yellow-white. Abdomen largely grey, with yellow patches on and below spiracular line, and yellowish prolegs.

Spines absent, except on abdominal segments 9 and 10, where they are replaced by unbranched, peg-shaped structures. Prothorax dorsally and anteriorly more strongly produced than in the feeding larva, giving it a hooded appearance.

**Identification**

Other spiny West Palaearctic Blennocampinae larvae belong to the genera *Monardis*, *Periclista*, *Pareophora*, *Monophadnoides* and *Claremontia*.  Larvae of all of these, none of which feeds on *Fraxinus*, have a mainly pale green or yellowish body and are thus easily distinguished from the predominantly grey larva of *E. exarmata*. Smith 1969 stated that the larva of *Eupareophora parca* has two bifurcate spines on the subspiracular lobe, and can therefore be distinguished from those of *Periclista* species in which the anterior of these two spines is bifurcate and the posterior one simple. The larva of *E. exarmata* in this respect (Fig. 4) is however like *Periclista*, not *E. parca*. The coloration of the mature larva of *E. parca* (illustrated by Williams 2007) and *E. exarmata* (Figs 2, 3, 4) is similar, although the latter is apparently darker. According to Williams 2007 (fig. 10B), the caudal abdominal terga of the *E. parca* prepupa entirely lack large cuticular processes, whereas the prepupa of *E. exarmata* clearly possesses some (Fig. 5).

**Behaviour**

All adult collection records and observations on larvae (here, and by Supatashvili et al. 1972, suggest that *E. exarmata* is univoltine, flying soon after bud-break in spring, with larvae developing, according to local climate, between the end of April and start of June. The behaviour of the feeding larvae, although only briefly described by Supatashvili et al. 1972 seems to resemble quite closely that of the Nearctic *E. parca* as described by Williams 2007.

At Hagen-Hohenlimburg only mature larvae of *E. exarmata* were found, apparently when they crawled down the trunk of the host in order to reach a spot in which to complete their development. Williams 2007 recovered eight prepupae of *E. parca* from the soil litter layer, and 2 from branches of the host. Further observations are therefore needed to establish whether *E. exarmata* always leaves its host before moulting to a prepupa. According to Supatashvili et al. 1972, the mature larvae form cells in the bark of *Fraxinus*, in which they overwinter. A further apparent peculiarity noted by these authors, is that the freshly moulted larvae lack spines, but that these re-appear within a day. Neither of these phenomena was observed by Williams 2007 in the Nearctic *E. parca*, who found that the rather flimsy cocoon was usually constructed in the upper layers of the soil.

## Supplementary Material

XML Treatment for Eupareophora exarmata
